# Physiological demands of racket sports: a systematic review

**DOI:** 10.3389/fpsyg.2023.1149295

**Published:** 2023-03-30

**Authors:** María Pía Cádiz Gallardo, Francisco Pradas de la Fuente, Alejandro Moreno-Azze, Luis Carrasco Páez

**Affiliations:** ^1^Faculty of Health and Sports Science, University of Zaragoza, Zaragoza, Spain; ^2^Training, Physical Activity and Sports Performance Research Group (ENFYRED), University of Zaragoza, Zaragoza, Spain; ^3^, BIOFANEX Research Group (CTS-972), Department of Physical Education and Sport, University of Seville, Sevilla, Spain

**Keywords:** heart rate, maximum oxygen consumption, lactate, padel, badminton, table tennis, tennis

## Abstract

The practice of racket sports has had an exponential growth in the last decade, along with it, the scientific interest in researching the different disciplines: badminton, padel, table tennis, tennis, and squash. However, most research has focused on the technical and tactical field. Therefore, the aim of this review is to analyze and compare the indicators of the internal load of each sport: heart rate (HR), maximum oxygen consumption (VO_2max_), oxygen consumption (VO_2_), and lactate (LA) in order to reset physiological references to adjust the training of the players and also use these references to propose the practice of these sports for healthy purposes to the general population. PRISMA Guidelines for Systematic Review were used to search for articles that met the inclusion criteria in three databases: Web of Science, Pubmed, and Sportdiscus. The search was performed between January 2010, and September 2022. Finally, a total 27 records were included for analysis in this study. The main findings were related to the differences in the intensity rates between sports. The highest lactate concentrations and heart values were found in badminton in the intensity of effort values (whose highest values were found in badminton) 10.11 (±4.99) mmol/L and 182.6 (±2.7) bpm respectively, whereas table tennis showed the lowest ones 1.2 (± 0.4) mmol/L, and 103.99 (±15.09) bpm, respectively. The highest mean VO_2_ was found in table tennis with a value of 36.8 (±13.2) ml/kg/min and the lowest in tennis with a value of 26.6 (±2.7) ml/kg/min. The highest VO_2max_ was found in tennis players 58.0 (±4.6) ml/kg/min, and the lowest value was in table tennis with a value of 42.9 (±4.2) ml/kg/min. Since most of the studies were carried out on elite men players, future research should focus on amateur and women level players.

## 1. Introduction

The so-called racket sports include a variety of disciplines such as: badminton, racquetball, padel, tennis, table tennis, squash, etc., which are competed individually or in pairs. Racket sports are characterized by being acyclical disciplines, which combine very intense physical load cycles with short breaks, allowing incomplete recovery from the efforts performed (Martínez, 2014).

In the last decade, racket sports have become an important alternative to traditionally practiced sports, considerably increasing the number of people who practice them. One of the main factors that differentiates each of the racket sports is the court on which the game takes place. Some of them are developed in spaces divided by a net (badminton, tennis, and table tennis); in others it is mandatory to use a wall or fence (squash and racquetball); and in the case of padel, a mixed sport developed in a space enclosed by side and back walls and divided by a net where it is allowed to play with some side wall areas and with the back walls.

Despite the importance that technical-tactical variables undoubtedly have in racket sports, since all the decisions made during the game determine performance and victory (Stare et al., 2015; Varas Caro and Gómez-Ruano, 2016; Cui et al., 2017), it is also interesting to analyze other aspects that have a direct influence on sports performance, such as the physiological response or the metabolic impact, indicators that inform us about the internal load, that it is to say variables of great relevance to know the intensity of the effort in a match.

These metabolic demands depend, mainly on the type of effort required in each sports specialty and the distribution of the periods of effort and rest established in each case. In this sense, considering the game elements of each discipline, such as scoring, as indicated by International Tennis Federation (2022) matches can be played to the best of three or five sets, whose duration can fluctuate between 1 to 5 h; although most matches are played to the best of three sets, with an average duration of 60 to 90 min (Bergeron et al., 1991; Torres-Luque et al., 2014). According to the rules of the International Padel Federation (2021), padel matches are played to the best of three sets whose duration according to the study carried out by Sánchez-Alcaraz (2014) was 35 min in men, and 36 min in women. As described above, padel and tennis have similar elements that are manifested through the type of scoring and rest times: 20 s between points, 90 s between side changes and 120 s between sets (Spanish Padel Federation, 2022), but they differ in the dimensions of the court (Amieba and Salinero, 2013) and absence of walls. Badminton has similar scoring characteristics to tennis and padel; in addition, the matches are developed to the best of three sets, according to the Badminton World Federation (2022), whose duration fluctuates between 17 and 40 min (Ming et al., 2008; Abian-Vicen et al., 2013). Squash matches usually take place over five sets, but sometimes also over three sets (World Squash Federation, 2020). Whereas in table tennis the score is different from the sports mentioned, since the matches are developed to the best of any odd number of games (Royal Spanish Table Tennis Federation, 2021–2022), and the duration of a match fluctuates between 10 to 25 min (Lees, 2003).

About resting times during racket sports competition, we can point out that in padel between each set are 2 min, 20 s maximum between each game, when changing side by game the maximum time is 1 min and 30 s, and in the case of tie-break the player has 20 s for the change of side (International Padel Federation, 2021). In tennis, the players have a maximum rest of 25 s between points. When players switch sides at the end of a game a maximum break of 1 min and 30 s allowed. However, after the first game of each set and during a tie-break game, the game is continuous, and players will switch sides without any rest. At the end of each set there is rest of a maximum of 2 min (International Tennis Federation, 2022). In table tennis the game is continuous throughout the match, except that every player is entitled to a break of up to 1 min between successive games of a match; short breaks to use the towel after every 6 points from the beginning of each game and in the change of side in the last possible game of a match. Moreover, a player or partner may request a timeout period of up to 1 min during a match (RFETM, 2021–2022). In badminton matches there is a break that should not exceed 1 min during each game when one side reaches 11 points; in addition, a break that should not exceed 2 min between the 1st and 2nd game, and between the 2nd and 3rd game in all matches (Spanish Badminton Federation, 2019). In squash the allowed rest time should not exceed 1 min and 30 s between the end of the warm-up and between each game (Badminton World Federation, 2022).

According to these resting, we can say that table tennis and squash are the sports that have less recovery time, unlike tennis, padel and badminton. This could impact the values of physiological markers used to analyze internal load.

Another factor that could intervene in the internal load are the types, amount, and frequency of strokes. In tennis, strokes such as serve, lobs, deep groundstrokes, are characterized by explosive and fast movements with high energy consumption in a short time (Kovacs, 2007). It also occurs in table tennis, with the difference that the speed and explosiveness with which the game is developed is much greater, finding differences between sexes being the number of strokes made by women than men (Torre et al., 2022). In badminton the strokes that are made during the game are also very fast, likewise, the speed of the technique with minimum preparation, the thinking speed and the risk they take in attacking blows (Stovba et al., 2020). Similarly, the strokes in padel resemble those of tennis since they share some rules. The difference relies in the alternating hits that can be made between both players in addition to being able to slow down the game using both the background walls and the side walls (García-Benítez et al., 2018). On the other hand, according to the description above, the punctuation systems are similar, but in the case of padel, a new rule incorporated in 2020, which is that if both teams have won three points, the score will be deemed “equal” and a single decisive point will be played, called “Golden Point.” The pair that wins the decisive point will win the game (World Padel Tour, 2022).

Additionally, the anthropometric characteristics of the players of each sport is another variable to considered. In badminton, according to the study conducted by Ramos Álvarez et al. (2016) women are ectomorphs and men mesoform. In padel, as stated by Pradas de la Fuente et al. (2015a) the anthropometric profile in women is endosomesomemorphic, because they present a greater development of skeletal muscle than fat. Endomorphic somatotypes were negatively related to performance, while ectomorphic profiles seems more effective. In table tennis, as reported by Pluta et al. (2021) and Pradas et al. (2021a) the predominant somatotype in men is mesomorphic, while ectomorphic in women. The study of Pradas et al. (2021a) showed that mototype was predominatly mesomorphic in men and endomorphic in woman. Higher leans-mass in the upper limbs appeared to be associated with better performance in table tennis player.

The court and the type of movements made are other aspects that might intervene in the physiological response and suggest some difference between each sport because of the difference in court sizes. Padel is played on a rectangular court of 20 × 1 0m with 10 m bottom walls, and side walls of 20 m (Pradas de la Fuente et al., 2015a). In the study conducted by García-Benítez et al. (2018) this size generates an improved pace of play and greater frequency of actions without increasing physical intensity compared to other sports, such as tennis where the playing field is a rectangle measuring 23.77 × 8.23 m, for singles. For doubles the court has the same sizes as singles, but the width is 10.97 m (International Tennis Federation, 2022). Table tennis is played in a rectangle measuring 2.74 × 1.525 m (ITTF, 2020), badminton is played in a rectangle too, that measures 13.40 × 5.18 for singles, and 13.40 × 6.10 (Badminton World Federation, 2022) for doubles.

The dimensions of the playing court of each sport have a direct relationship with the physiological and metabolic response since the path of these are different in each sport. For example, in padel, 52.32% of the total number of strokes are lateral displacements and 42.29% are frontal (Priego et al., 2013), while in the case of table tennis a playing surface of such small dimensions causes short and fast very explosive high intensity movements although the final distance traveled is short but its intensity is very high (Torre et al., 2022). Similarly occurs with badminton, but on a court of larger dimensions as in tennis (Kovacs, 2007; Stovba et al., 2020). This aspect has a direct relationship with the density of play (activity time//rest time).

About the temporal structure game, according to the study of Pradas et al. (2021a) table tennis with other racket sports, the playing times are longer in badminton with values of 6.8 s in men competition and 2.3 s in women competition (Fernandez-Fernandez et al., 2007). In tennis, the duration of points is longer than in table tennis, with playing times 5.2, s for men and 7.1 s for women (O'Donoghue and Ingram, 2001). In padel the duration is also more than in table tennis with effort values 9.3 and 17.7 s 9.06 and 13.03 in women (Pradas de la Fuente et al., 2015a).

As described above, here are multiple factors involved in the physiological response, such as the dynamics of the game, the type of surface, the dimensions of the court, the distances traveled, the breaks in each match and the type of effort made, which in some game actions will predominate the efforts of resistance, strength, and speed (Montoya et al., 2020). This variability of efforts will cause different metabolic and physiological responses in athletes, due to the interval nature of these sports (Sánchez-Alcaraz, 2014). To evaluate these responses, different markers have been assessed, being blood lactate concentrations (LA), heart rate (HR) and oxygen consumption (VO_2_) the most used (Carrasco et al., 2011; Kondrič et al., 2013).

Available research in racket sports mainly refers to the structure of the game and how the different technical-tactical factors are involved in performance (Courel-Ibañez et al., 2017; Fernández-García et al., 2020; Navas et al., 2020; Ramón-Llin et al., 2020). However, in relation to physiological responses we can find a diversity of results in studies of performance in rackets sports, considering all the elements of the game involved in sports performance and how they determine the response of the player’s body during a match. According to the above and considering the growth that racket sports have had in recent years, the aim of this review is to analyze and compare the indicators of the internal load of each sport: HR, VO_2max_, VO_2_, and LA in order to reset physiological references better adjust the training of the players and also use these references in order to propose the practice of these sports for healthy purposes to the general population.

## 2. Materials and methods

For the performance of this review, the protocol was registered in PROSPERO 2022 CRD42022354791, and the PRISMA Guidelines for Systematic Reviews was used (Page et al., 2021). The search was carried out in the following databases: Web of Science, Sportdiscus and Pubmed, in all fields, using MeSH and Thesauri terms derived from the rackets sports.

The search strategy used the following terms:

- Racket sports:” racquet sports” OR “racket sports” OR “racket players” OR Badminton OR “Badminton players” OR squash OR “squash sport” OR padel OR “paddle tennis” OR paddle OR “padel players” OR tennis OR “Tennis player” OR “Table Tennis” OR “ping pong” OR “table tennis players.”
- Exercise intensity: “exercise physiology” OR “exercise intensity” OR “clinical exercise physiology.”
- Physiological markers: “Lactic Acid” OR “Lactate” OR “Lactates” OR “BLOOD lactate” OR “heart rate” OR “Heart rates” OR “Cardiac Rate” OR “rate heart” OR “heart rate monitoring” OR “heart rate monitors” OR “oxygen consumption” OR “VO_2_ peak” OR “VO_2max_” OR “exercise physiology” OR “exercise intensity” OR “clinical exercise physiology.”
- Sintax: (“racquet sports” OR “racket sports” OR “racket players” OR Badminton OR “Badminton players” OR squash OR “squash sport” OR padel OR “paddle tennis” OR paddle OR “padel players” OR tennis OR “Tennis player” OR “Table Tennis” OR “ping pong” OR “table tennis players”) AND (“Lactic Acid” OR “Lactate” OR “Lactates” OR “blood lactate” OR “heart rate” OR “Heart rates” OR “Cardiac Rate” OR “rate heart” OR “heart rate monitoring” OR “heart rate monitors” OR “oxygen consumption” OR “VO_2_ peak” OR “VO_2max_” OR “exercise physiology” OR “exercise intensity” OR “clinical exercise physiology”)

For the selection of studies, the following criteria were considered: articles published between 2010 and September 2022, articles in English and Spanish, studies in which adult male and female players (18 years or older) were analyzed in real competition or in simulated situations, and studies that assessed the following physiological variables: maximum oxygen consumption, heart rate, lactate and others derived from the above and articles published in full-text academic journals.

On the other hand, the exclusion criteria were the following: systematic reviews, narrative reviews meta-analysis, scoping reviews, articles that analyzed the adapted sport, articles that analyzed subjects with different pathologies, abstracts, conferences and/or communications to congress.

Once the search strategy was applied in the databases the selection bias was controlled for by the two researchers who used the Endnote web manager (available online at https://www.myendnoteweb.com/EndNoteWeb.html) bibliographic manager. This software was also used to identify duplicates once the main search was conducted. For quality bias, each investigator independently applied the National Heart, Lung, and Blood Institute (NHLBI) Quality Assessment Tool for Short, Cross-Sectional Observational Studies available online https://www.nhlbi.nih.gov/health-topics/study-quality-assessment-tools; and for the case studies the tool available online was applied https://www.nhlbi.nih.gov/health-topics/study-quality-assessment-tools. The document is organized by number of questions to assess the quality of the studies, registering three indicators, as appropriate: “Yes” (Y), “No” (N), or “Other” (O): “cannot determine” (CD); “not applicable” (NA); “not reported” (NR).

The application of this tool was based on the study design, and it was used for the general measuring of the quality of the studies: “good,” “fair” and “poor.” Two researchers applied this tool independently and in case of disagreement, a consensus was reached through discussion, or the opinion of a third researcher was requested.

The Supplementary Table 1 have the results of the evaluation.

## 3. Results

**Figure 1:**
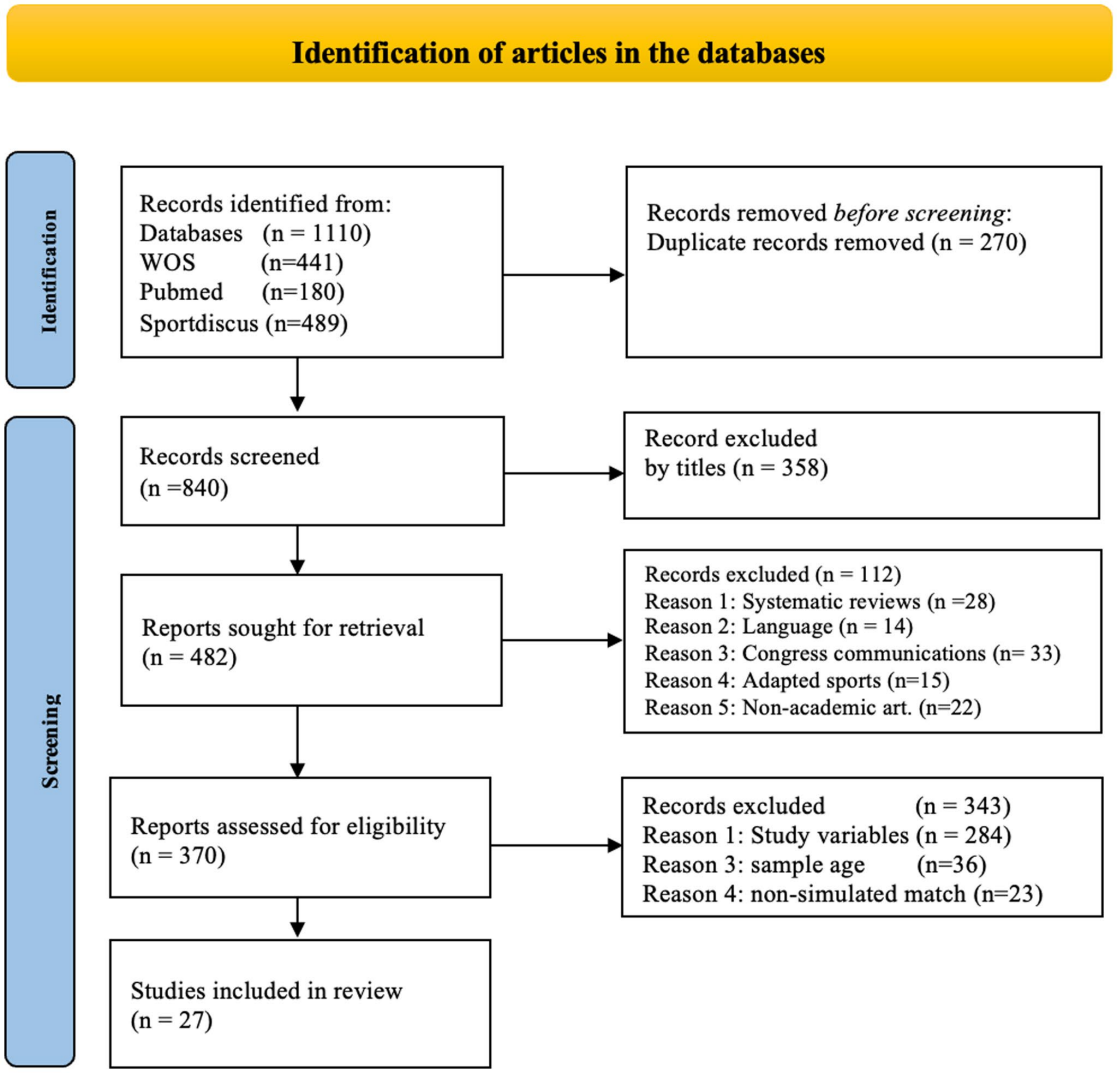
Flow chart of the search process the selection of article.

The initial search yielded a total of 1,110 articles. After removing duplicates, a total of 840 articles were reviewed. Once the inclusion and exclusion were applied criteria 27 articles were selected for the final analysis (Figure 1).

According to the results obtained in the evaluation of the quality of the studies, the risk of bias is negative, since most of the studies respond to good quality.

The selected studies were organized by sport to present the results of each physiological marker. In table tennis 8 studies were found, 6 in tennis, 6 in padel and 7 in badminton. Surprisingly, no studies in squash.

The most used parameter to analyze the internal load was HR, since all studies selected 27 studies analyzed this variable. In relation to concentration LA, this variable was analyzed in 14 studies, VO_2max_ was analyzed in 12 studies and VO_2_ in 6 studies.

The studies found involved female and male subjects, and there were some studies whose sample was mixed.

The selected studies analyzed variables in a diversity of subjects with different levels of play. Below are the levels found.

The values found in each study were organized by each sport to present the number of subjects, age, intervention, mean LA, VO_2max_, VO_2_, and HR.

## 4. Discussion

The main objective of this review was to analyze the indicators of internal load to reset physiological references to adjust the training of the players and suggest the practice of the general population.

According to the search strategy and the inclusion and exclusion criteria applied, the sports selected for this review were tennis, table tennis, padel and badminton. The findings found in the studies of these sports refer to the analysis of internal load using four main physiological markers: HR, VO_2max_, VO_2_ and LA, being HR the most used.

### 4.1. Maximum oxygen consumption (VO_2max_) and oxygen consumption (VO_2_)

VO_2max_ is the ability to transport and consume oxygen during strenuous work; it is associated with cardiorespiratory fitness and is used as an indicator in this field; it measures aerobic capacity and, therefore, defines the limits of cardiovascular function (Koutlianos et al., 2013; Sánchez-Otero et al., 2014).

In table tennis of the 7 included studies, 5 evaluated VO_2max_ (Shieh et al., 2010; Zagatto et al., 2016; Milioni et al., 2018; Pradas et al., 2021b; Torre et al., 2022) these analyses correspond to the study carried out on a men sample, whose values fluctuated between 42.1 (±6.4) and 53.2 (±0.6) ml/kg/min.

The variable VO_2_ was analyzed by only 3 studies (Shieh et al., 2010; Zagatto et al., 2016; Milioni et al., 2018), whose values fluctuated between 28.5 (±3.02) and 35.6 (±18.4) ml/kg/min. The cause of shortages of analysis of this variable could be associated with the complexity involved in carrying a device when playing a simulated match, and even more so in an official match.

In tennis, of the 6 included studies, only 3 evaluated VO_2max_ (Baiget et al., 2015; Kilit et al., 2016; Hoppe et al., 2020) the values fluctuated between 40.9 (±4.3) and 58.0 (±4.6) ml/kg/min, in women and men, respectively. The mean value the VO_2_ in play fluctuated between 26.6 (±2.7) and 29.9 (±3.7) ml/kg/min. These values correspond to the group of men. These values are similar to those presented in other studies (Smekal et al., 2001; Ferrauti et al., 2003) using game simulation and real competition situations.

In badminton, one study analyzed the VO_2max_ in a sample whose level of play was recreational (Deka et al., 2017), the value was found to be 45.2 (±8.7) ml/kg/min, while the meanVO_2_ recorded during the game was 34.4 (±5.8) ml/kg/min, values very similar to those found in another study (46.0 ± 4.5 ml/kg/min; Faude et al., 2007), but the sample analyzed was an elite level group.

In the case of padel 2 studies recorded the VO_2max_ (Pradas de la Fuente et al. 2015a; García et al., 2017), where the values fluctuated between 47.33 (±4.57) and 51.15 (±5.73) ml/kg/min. The VO_2_ during a match was not recorded by any studies of this sport. However, in one study (Carrasco et al., 2011) the variable analyzed in young players the VO_2máx_ was 55.64 (±8.84) ml/kg/min, and the mean value of VO_2_ in matches was 24.06 (±6.95) ml/kg/min.

The highest VO_2max_ values reported was 58 (±4.06) ml/kg/min (Baiget et al., 2015) corresponding to tennis and the lowest value was 42.1 (±6.4) ml/kg/min (Shieh et al., 2010) corresponding to table tennis. In a study we found an intermediate value (Deka et al., 2017), whose value was 45.2 (±8.7) ml/kg/min in a recreational sample. These values are similar to the results obtained in a study in national game players (Milioni et al., 2018). Although, there are similar values among national and recreational players in badminton and table tennis the difference is that players of national category can tolerate much greater physical efforts (Fernandez-Rodriguez et al., 2019).

The maximum VO_2_ during a match was 36.8 (±13.2) ml/kg/min registered in one study (Shieh et al., 2010), focused on table tennis, whereas the minimum value recorded in matches was 26.6 (±2.7) ml/kg/min registered in tennis (Kilit et al., 2016).

However, and according to the above results we could say that VO_2_ does not seem to be a limitation for racket sports practice (Mellado-Arbelo and Baiget, 2022).

### 4.2. Heart rate

Of the totality of the studies selected for this review, this physiological marker was analyzed by 25 studies, which shows that it is one of the most used markers to measure the intensity of effort (Kondrič et al., 2013). This could be due to the easy access to heart monitors, and the low interference generated by the device in the performance of the players in match.

In table tennis, 7 studies analyzed the HR as variable (Martin et al., 2015; Pradas de la Fuente et al., 2015a; Zagatto et al., 2016; Milioni et al., 2018; Picabea et al., 2021; Pradas et al., 2021b; Torre et al., 2022) whose mean values fluctuated between 103.9 (±15.09) and 146 (±5.9) bpm. In a study conducted in a group of young players of competitive level (Sperlich et al., 2011), the mean HR in a simulated match was 125 (±22) bpm.

In tennis, of the 6 studies found, 5 analyzed this variable (Gomes et al., 2011; Martin et al., 2011; Baiget et al., 2015; Kilit et al., 2016; Hoppe et al., 2020), where the values fluctuated between 128 bpm and 154 bpm.

The values found in this sport were found in elite levels of players, however this data could be a reference for recreational players.

In badminton all selected studies analyzed HR, whose values fluctuated between 157 (±13.9) and 182 (±9.4) bpm these results indicate that the intensity of this type of sports is moderate to vigorous, because of the high averages recorded during the match (Cabello Manrique and González-Badillo, 2003; Faude et al., 2007).

The highest value was found in the study conducted in a sample of competitive national level players, mean HR was the 182.6 (±2.7) bpm (Chen et al., 2011). These results may be associated with the characteristics of this sport, since it is considered one of the three most competitive, intense and fastest racket sports (Stovba et al., 2020).

In padel, this variable was analyzed in all studies, fluctuating between 126.7 and 159.1 bpm. Two studies (Castillo-Rodríguez et al., 2014; Roldán-Márquez et al., 2022) analyzed this variable in national level players, reporting values between 135 (±7.9) and 149.1 (±18.2) bpm, respectively. The last value is similar to that recorded in the research by Carrasco et al. (2011), who found a mean HR of 148.3 (±13.63) bpm in a sample of first-level juniors players. The highest mean value found was in badminton with a value of 182.6 (±2.7) bmp registered in one study (Chen et al., 2011), and the lowest value was found in table tennis (mean HR of 103.99 (±15.09) bpm; Picabea et al., 2021). It is well known that racket sports are characterized by being sports of variable intensity. HR values reported suggest that tennis, table tennis and padel dominate the aerobic pathway, with very short high intensity intervals. In the case of badminton, the values found suggest that the dominant route is anaerobic.

### 4.3. Lactic acid concentrations

In table tennis, of the 8 studies found, 6 analyzed this variable (Martin et al., 2015; Pradas de la Fuente et al., 2015b; Zagatto et al., 2016; Milioni et al., 2018; Pradas et al., 2021b; Torre et al., 2022), LA concentrations ranged between fluctuated 1.2 (±0.4) and 4.7 (±2.2) mmol/L. The highest value found in the one study (Martin et al., 2015) was because the blood sample taken was 1 min after the end of each set and 3 min after the end of the matches to respect International Table Tennis Federation timing rules. However, in the others studies blood sample was taken after the end of each set, and in the 3^er^, 5^th^, and 7^th^ min after the match; this explains the differences found.

Concentrations found in table tennis are less than 2 mmol/L, so it could be said that the dominant metabolic pathway is aerobic, with little participation of the anaerobic lactic pathway (Zagatto et al., 2018).

In tennis, of the 6 selected studies, 4 analyzed this marker, 2 studies were performed on men and two studies on women (Mendez-Villanueva et al., 2007; Gomes et al., 2011; Martin et al., 2011; Hoppe et al., 2020). The values recorded in these studies fluctuated between 1.5 and 5.7 (±1.8) mmol/L, being similar to those found in other studies (Fernandez-Fernandez et al., 2007).

In the study where the values in women and men were analyzed, and compared according to the type of court (Martin et al., 2011), the highest value was found in women whose game was developed in clay courts. In addition to the differences between gender, it seems that the type of court could interfere with the dynamics of the game and consequently in the metabolic response, an aspect that should be considered in future research for the analysis of the internal load. In the studies conducted in women, the highest value found was 5.7 (±1.8) mmol/L recorded in a clay court, whereas the lower value was 1.5–2.3 mmol/L. This difference could be associated with the players at competitive level since the highest value corresponds to elite players and the lowest to national players. In more demanding game situations LA concentrations could rise between 6 and 8 mmol/L (Fernandez-Fernandez et al., 2007; Mendez-Villanueva et al., 2007; Christmass et al., 2010).

In badminton, of the 7 studies found, 2 analyzed circulating levels of LA, whose recorded values fluctuated between 4.3 (±0.4) and 10.1 (±4.9) mmol/L, although the highest value corresponds to the analysis in a recreational male player; this value is like that recorded in other study (Mendez-Villanueva et al., 2007), whose values are related to long-lasting matches. According to the studies found, we can say that most focus on elite level players (Patterson et al., 2017). However, the values found suggest that the dominant metabolic pathway is glycolytic anaerobic.

In padel, of the 6 studies found, only 3 analyzed the LA concentrations (Castillo-Rodríguez et al., 2014; Pradas de la Fuente et al., 2015a; Roldán-Márquez et al., 2022). The recorded values fluctuated between 1.83 and 2.87 mmol/L. Blood LA levels were ower than those found in studies of badminton and tennis, therefore, it could be estimated and according to the characteristics of this sport (Pradas et al., 2021a) that the metabolic pathway is aerobic due to the long duration of the matches, and at the same time anaerobic due to the actions that take place in the game, such as changes of direction and accelerations (Courel-Ibáñez and Herrera-Gálvez, 2020). Racket sports are characterized by being cyclical in nature, but for training to have positive effects on the game, technical characteristics, tactics, age, sex, level of play must also be considered, and in the case of tennis, the type of court. It is important to consider all the factors mentioned above since they are involved in the physiological response.

According to the variables analyzed in this systematic review, we could say that badminton is the most physiologically demanding sport, this could be due to the important technical demand during the game that is characterized by jumping, changes of direction and acceleration (Cabello Manrique and González-Badillo, 2003). This could also be due to the duration of a game point, technical characteristics, or rest periods between one game and another. The value found in LA concentration in this review is far from the study carried out before the last decade (Majumdar, 1997) whose LA concentrations were 4.7 mmol/L in real match conditions. The high values of the FC may be characteristics of the game. It requires efforts of high intensity and short duration that generate a high level of stress, with the consequent stimulation of the sympathetic nervous system and as a consequence the increase of HR (Ramos Álvarez et al., 2016) In addition, all the load is distributed in the different muscle groups and in all body systems, therefore, they create an important biological reserve in high performance (Stovba et al., 2020). In table tennis, the values found in this study suggest could be related to the characteristics of the game, explosive and very fast (Torre et al., 2022). In response to these characteristics it seems that the main energy pathways are aerobic and anaerobic, because is a sport characterized by skill rather than VO_2_, were fast and intense actions predominant. The mean values of VO_2_ are lower than badminton; his could be due to the high technical demand, the short duration of the points and the shorter routes that characterize badminton. This could be explained the metabolic and cardiorespiratory demands are moderate throughout the match. Moreover, as explained by Sperlich et al. (2011) metabolic and cardiorespiratory demands are moderate throughout the match. In t the mean values of VO_2_ are lower than badminton; his could be due to the high technical demand, the short duration of the points and the shorter routes that characterize badminton.

In the case of padel according to the results analyzed it could be suggested that the most used metabolic pathway is glycolytic aerobic, lactate values show that the energy coming from the lactic anaerobic system is not predominant. In this sense, we found similar results in another study (Pradas de la Fuente et al., 2015a) where the maximum value obtained was close to 2.4 mmol/L, indicating that the glycolytic anaerobic pathway is the least determining. Another study shows that mean values transit from aerobic-anaerobic zone. This occurs because it is an intermittent sport, whose highest HR occurs in games and the lowest in breaks (Muñoz et al., 2018). In tennis, according to the results obtained, it could be said to be a sport with lower physiological demands than badminton. This could be explained by the recovery phases (Edel et al., 2019), and the speed of gaming actions. According to the study of Kovacs, (2007) the main contributor is the anaerobic ATP-PC, since it is a source of immediate energy that is used mainly in some hits, such as in serves at more than 200 km/h. However, in the study by Bergeron et al. (1991) it was found that the overall metabolic response is prolonged exercise of moderate intensity. It appears that oxidative metabolism restores ATP for the duration of a full match due to recovery periods (Kerr, 2015). It seems that the energy systems for to racket sports are the aerobic and anaerobic (Simpson et al., 2017).

According to the findings, it is necessary to assess whether badminton is a recommended discipline for the general population, given the characteristics of the intensity of the effort manifested in the values found.

The limitations of this review were the scarcity of research conducted on women, since most focused on men, also the type of sample since the most studies were carried out in elite o competitive players. There are very few studies conducted at amateur or recreational levels of play. And additionally, the lack of observational studies.

Likewise, we did not find any studies of squash in the period that this research was carried out, except for before the last decade (Vučković et al., 2013). This highlights the need to generate more studies on the female gender, at different levels of play, considering, in the case of tennis, the type of court. In addition, generate studies on the effects of a physical exercise program based on one or more racket sports on the health status of different populations of interest. Given that the practice of these sports has been growing in recent years, both at a competitive and recreational level, it is necessary to know the physiological requirements.

## 5. Conclusion

Racket sports are disciplines characterized by being intermittent, however according to the results found in this review we find some differences between one sport and another. It can be said that badminton is the sport with the highest cardiovascular and metabolic demand, since we find the highest values in HR and LA; the latter exceeds the limit of the established aerobic and anaerobic threshold (2–4 mmol/L). In table tennis the values of HR and LA are lower than those of badminton, but the average value of VO_2_ is the highest of all the sports analyzed; this could be because specific skills are the most determining factor for the performance of this racket sports. In tennis, VO_2_ was the lowest value found, it seems that cardiovascular capacity would not be a limitation to practice this sport. In padel the values are lower than badminton, tennis and table tennis; the results suggest the dominant pathway could be aerobic with very short high intensity intervals.

The analysis of the physiological markers was made in samples with different levels of play (elite, professional, amateur, recreational) despite the well-known differences, it could be that padel, table tennis, tennis and badminton are recommended for general practice for healthy purposes, the latter being the most demanding of all.

Future research should focus on women, as most studies are conducted on men. Also, considering the growth of the practice of these sports, future research should include amateur or recreational level players with the purpose of knowing the effects on health.

## Author contributions

MC, FP, LC, and AM-A: research concept and study design. MC, FP, and LC: literature and review and write of the manuscript and conceptualization and methodology, formal analysis investigation, and resources data analysis. All authors contributed to the article and approved the submitted version.

## Conflict of interest

The authors declare that the research was conducted in the absence of any commercial or financial relationships that could be construed as a potential conflict of interest.

## Publisher’s note

All claims expressed in this article are solely those of the authors and do not necessarily represent those of their affiliated organizations, or those of the publisher, the editors and the reviewers. Any product that may be evaluated in this article, or claim that may be made by its manufacturer, is not guaranteed or endorsed by the publisher.
