# Supplementary material for: Physiological demands of racket sports: a systematic review
Source: Front Psychol. 2023 Mar 30;14:1149295. doi: 10.3389/fpsyg.2023.1149295 (PMC10101231; doi:10.3389/fpsyg.2023.1149295)
Supplement: Supplementary file 1 [file Table_1.docx]

Supplementary Material

***PHYSIOLOGICAL DEMANDS OF RACKET SPORTS***

***A SYSTEMATIC REVIEW***

María Pía Cádiz Gallardo, Francisco Pradas de la Fuente*, Alejandro Moreno-Azze, Luis Carrasco Páez.

*** Correspondence:** franprad@unizar.es

**Table 1.** Results of quality assessment of the NHI tool for observational cohort and cross-sectional studies.

| Table Tennis | | | | | | | | | | | | | | | | | |
| --- | --- | --- | --- | --- | --- | --- | --- | --- | --- | --- | --- | --- | --- | --- | --- | --- | --- |
| Author | Year | | 1 | 2 | 3 | 4 | 5 | 6 | 7 | 8 | 9 | 10 | 11 | 12 | 13 | 14 | Score |
| Martin et al | 2015 | | Y | Y | Y | Y | Y | N | N | NA | Y | NA | Y | N | NA | N | good |
| Milioni et al | 2018 | | Y | Y | Y | Y | Y | N | N | NA | Y | NA | Y | N | NA | N | good |
| Picabea et al | 2021 | | Y | Y | Y | Y | Y | N | N | NA | Y | NA | Y | N | NA | N | good |
| Pradas de la Fuente et al | 2015b | | Y | Y | Y | Y | NR | N | N | NA | Y | NA | Y | N | NA | N | fair |
| Pradas et al | 2021b | | Y | Y | Y | Y | Y | N | N | NA | Y | NA | Y | N | NA | N | good |
| Shieh et al | 2010 | | Y | Y | Y | Y | NR | N | N | NA | Y | NA | Y | N | NA | N | fair |
| Torre et al | 2022 | | Y | Y | Y | Y | Y | N | N | NA | Y | NA | Y | N | NA | N | good |
| Zagatto et al | 2016 | | Y | Y | Y | Y | Y | N | N | NA | Y | NA | Y | N | NA | N | good |
| Tennis | | | | | | | | | | | | | | | | | |
| Author | Year | | 1 | 2 | 3 | 4 | 5 | 6 | 7 | 8 | 9 | 10 | 11 | 12 | 13 | 14 | Score |
| Baiget et al | 2015 | | Y | Y | Y | Y | Y | N | N | NA | Y | NA | Y | N | NA | N | good |
| Hoope et al | 2020 | | Y | Y | Y | Y | NR | N | N | NA | Y | NA | Y | N | NA |  | good |
| Kilit et al | 2016 | | Y | Y | Y | Y | NR | N | N | NA | Y | NA | Y | N | NA | N | good |
| Martin et al | 2011 | | Y | Y | Y | Y | NR | N | N | NA | Y | NA | Y | N | NA | N | good |
| Mendez-Villanueva et al | 2010 | | Y | Y | Y | Y | NR | N | N | NA | Y | NA | Y | N | NA | N | good |
| Badminton | | | | | | | | | | | | | | | | | |
| Author | Year | | 1 | 2 | 3 | 4 | 5 | 6 | 7 | 8 | 9 | 10 | 11 | 12 | 13 | 14 | Score |
| Abudalli et al | 2019 | | Y | Y | Y | Y | Y | N | N | NA | Y | NA | Y | N | NA | N | good |
| Bisschoff et al | 2016a | | Y | Y | Y | Y | Y | N | N | NA | Y | NA | Y | N | NA | N | good |
| Bisschoff et al | 2016b | | Y | Y | Y | Y | Y | N | N | NA | Y | NA | Y | N | NA | N | good |
| Bisschoff et al | 2018 | | Y | Y | Y | Y | Y | N | N | NA | Y | NA | Y | N | NA | N | good |
| Chen et al | 2011 | | Y | Y | Y | Y | Y | N | N | NA | Y | NA | Y | N | NA | N | good |
| Deka et al | 2017 | | Y | Y | Y | Y | Y | N | N | NA | Y | NA | Y | N | NA | N | good |
| Savarirajan et al | 2016 | | Y | Y | Y | Y | Y | N | N | NA | Y | NA | Y | N | NA | N | good |
| Padel | | | | | | | | | | | | | | | | | |
| Author | | Year | 1 | 2 | 3 | 4 | 5 | 6 | 7 | 8 | 9 | 10 | 11 | 12 | 13 | 14 | Score |
| Carbonell Martínez et al | | 2017 | Y | Y | Y | Y | Y | N | N | NA | Y | NA | Y | N | NA | N | good |
| Castillo-Rodriguez et al | | 2014 | Y | Y | Y | Y | Y | N | N | NA | Y | NA | Y | N | NA | N | good |
| García et al | | 2017 | Y | Y | Y | Y | Y | N | N | NA | Y | NA | Y | N | NA | N | good |
| Pradas de la Fuente et al | | 2015a | Y | Y | Y | Y | Y | N | N | NA | Y | NA | Y | N | NA | N | good |
| Ramón-Llin et al | | 2018 | Y | Y | Y | Y | Y | N | N | NA | Y | NA | Y | N | NA | N | good |
| Roldán-Márquez | | 2022 | Y | Y | Y | Y | Y | N | N | NA | Y | NA | Y | N | NA | N | good |

Quality of included studies was assessment using The National Institute (NHI) Quality Assessment tool for Observational Cohort and Cross-Sectional Studies https://www.nhlbi.nih.gov/health-topics/study-quality-assessment-tools. 1= Was the research question or objective in this paper clearly stated? 2= Was the study population clearly specified and defined? 3= Was the participation rate of eligible persons at least 50%? 4= Were all the subjects selected or recruited from the same or similar populations (including the same time period)? Were inclusion and exclusion criteria for being in the study prespecified and applied uniformly to all participants?; 5= Was a sample size justification, power description, or variance and effect estimates provided?; 6= ¿For the analyses in this paper, were the exposure(s) of interest measured prior to the outcome(s) being measured?; 7= Was the timeframe sufficient so that one could reasonably expect to see an association between exposure and outcome if it existed?; 8=For exposures that can vary in amount or level, did the study examine different levels of the exposure as related to the outcome (e.g., categories of exposure, or exposure measured as continuous variable)?; 9=Were the exposure measures (independent variables) clearly defined, valid, reliable, and implemented consistently across all study participants?;10=Was the exposure(s) assessed more than once over time?; 11=Were the outcome measures (dependent variables) clearly defined, valid, reliable, and implemented consistently across all study participants?; 12= Were the outcome assessors blinded to the exposure status of participants?; 13=Was loss to follow-up after baseline 20% or less?; 14=Were key potential confounding variables measured and adjusted statistically for their impact on the relationship between exposure(s) and outcome(s)?

**Additional references:**

Abdullahi, Y., Coetzee, B., and van den Berg, L. (2019) Relationships between results of an internal and external match load determining method in male, singles badminton players. *Journal of strength and conditioning research*, 33(4), 1111–1118. <https://doi.org/10.1519/JSC.0000000000002115>

Bisschoff, C., Coetzee, B., Esco, M. (2016a). Relationship between autonomic markers of Heart Rate and Subjective Indicators of Recovery Status in Male, Elite Badminton Players. *Journal of sports science & medicine*, 15(4), 658–669.

Bisschoff, C., Coetzee, B., Esco, M. (2016b) Relationship between heart rate, heart rate variability, heart rate recovery and global positioning system determined match characteristics of male, elite, African badminton player. *International Journal of Performance Analysis in Sport,* 16, 881-897. https://doi.org/ [10.1080/24748668.2016.11868936](http://dx.doi.org/10.1080/24748668.2016.11868936)

Bisschoff, C., Coetzee, B., Esco, M. (2018) Heart rate variability and recovery as predictors of elite, African, male badminton players performance levels. *International Journal of Performance Analysis in Sport*. [https://doi.org/18. 1-16. 10.1080/24748668.2018.1437868](https://doi.org/18.%201-16.%2010.1080/24748668.2018.1437868)

Carbonell Martínez, J. A., Ferrándiz Moreno, J. y Pascual Verdú, N. (2017) Análisis de la frecuencia cardíaca en el pádel femenino amateur (Analysis of heart rate in amateur female padel), Retos. 204-207. 10.47197/retos.v0i32.56040

Savarirajan, R. (2016) Result of heart rate, playing time and performance of tamilnadu badminton senior ranking players. *International Journal of Sports Sciences and Fitness*, 6(1), pp. 43–57.
