# Supplementary material for: Physiological demands of racket sports: a systematic review
Source: Front Psychol. 2023 Mar 30;14:1149295. doi: 10.3389/fpsyg.2023.1149295 (PMC10101231; doi:10.3389/fpsyg.2023.1149295)
Supplement: Supplementary file 2 [file Table_2.docx]

Supplementary Material

***PHYSIOLOGICAL DEMANDS OF RACKET SPORTS***

***A SYSTEMATIC REVIEW***

María Pía Cádiz Gallardo, Francisco Pradas de la Fuente*, Alejandro Moreno-Azze, Luis Carrasco Páez.

*** Correspondence:** franprad@unizar.es

**Table 2.** Results of quality assessment of the NHI tool for case-control studies.

| Author | Year | 1 | 2 | 3 | 4 | 5 | 6 | 7 | 8 | 9 | 10 | 11 | 12 | Score |
| --- | --- | --- | --- | --- | --- | --- | --- | --- | --- | --- | --- | --- | --- | --- |
| Gomez et al | 2011 | Y | Y | NR | NR | NA | NR | N | NA | NA | Y | NR | NR | poor |

Quality of included studies was assessment using The National Institute (NHI) Quality Assessment tool for Case-Control Studies https://www.nhlbi.nih.gov/health-topics/study-quality-assessment-tools.1=What the research question or objective in this paper clearly stated and appropriate? 2=Was the study population clearly specified and defined? 3=Did the authors include a sample size justification?; 4= Were controls selected or recruited from the same or similar population that gave rise to the cases (including the same timeframe)?; 5= Were the definitions, inclusion and exclusion criteria, algorithms or processes used to identify or select cases and controls valid, reliable, and implemented consistently across all study participants?; 6= Were the cases clearly defined and differentiated from controls?; 7= If less than 100 percent of eligible cases and/or controls were selected for the study, were the cases and/or controls randomly selected from those eligible?; 8= Was there use of concurrent controls?; 9=Were the investigators able to confirm that the exposure/risk occurred prior to the development of the condition or event that defined a participant as a case?; 10**=**Were the measures of exposure/risk clearly defined, valid, reliable, and implemented consistently (including the same time period) across all study participants?;11=Were the assessors of exposure/risk blinded to the case or control status of participants?; 12=Were key potential confounding variables measured and adjusted statistically in the analyses? If matching was used, did the investigators account for matching during study analysis?
