# Supplementary material for: Physiological demands of racket sports: a systematic review
Source: Front Psychol. 2023 Mar 30;14:1149295. doi: 10.3389/fpsyg.2023.1149295 (PMC10101231; doi:10.3389/fpsyg.2023.1149295)
Supplement: Supplementary file 3 [file Table_3.docx]

Supplementary Material

***PHYSIOLOGICAL DEMANDS OF RACKET SPORTS***

***A SYSTEMATIC REVIEW***

María Pía Cádiz Gallardo, Francisco Pradas de la Fuente*, Alejandro Moreno-Azze, Luis Carrasco Páez.

*** Correspondence:** franprad@unizar.es

**Table 3:** Number of studies according to sex of the sample.

| Sport | Men | Women | Mixed* | Total |
| --- | --- | --- | --- | --- |
| Table Tennis | 6(n=133) | 0 | 2(n=48 M/48F) | 8(n=229) |
| Tennis | 4(n=40) | 1(n=12) | 1(n=2M/6F) | 6(n=60) |
| Badminton | 6(n=111) | 0 | 1(n=10M/1F) | 7(n=122) |
| Padel | 4(n=70) | 2(n=15) | 0 | 6(n=85) |
| N° Studies | 20 | 3 | 4 | 27(n=496) |

*Studies whose sample were women and men.
