# Supplementary material for: Physiological demands of racket sports: a systematic review
Source: Front Psychol. 2023 Mar 30;14:1149295. doi: 10.3389/fpsyg.2023.1149295 (PMC10101231; doi:10.3389/fpsyg.2023.1149295)
Supplement: Supplementary file 4 [file Table_4.docx]

Supplementary Material

***PHYSIOLOGICAL DEMANDS OF RACKET SPORTS***

***A SYSTEMATIC REVIEW***

María Pía Cádiz Gallardo, Francisco Pradas de la Fuente*, Alejandro Moreno-Azze, Luis Carrasco Páez.

*** Correspondence:** franprad@unizar.es

**Table 4:** Number of studies according to level of players.

| Sport | Elite | Semiprofessional /Amateur | National/Elite | National | Amateur  /Elite | Professional | Recreational |
| --- | --- | --- | --- | --- | --- | --- | --- |
| Table tennis | 4 |  | 1 | 2 | 1 |  |  |
| Tennis | 3 |  |  | 1 |  | 2 |  |
| Badminton | 3 |  |  | 3 |  |  | 1 |
| Padel | 1 | 1 | 1 | 3 |  |  |  |
| Total studies | 11 | 1 | 2 | 9 | 1 | 2 | 1 |
