# Supplementary material for: Physiological demands of racket sports: a systematic review
Source: Front Psychol. 2023 Mar 30;14:1149295. doi: 10.3389/fpsyg.2023.1149295 (PMC10101231; doi:10.3389/fpsyg.2023.1149295)
Supplement: Supplementary file 5 [file Table_5.docx]

Supplementary Material

***PHYSIOLOGICAL DEMANDS OF RACKET SPORTS***

***A SYSTEMATIC REVIEW***

María Pía Cádiz Gallardo, Francisco Pradas de la Fuente*, Alejandro Moreno-Azze, Luis Carrasco Páez.

*** Correspondence:** franprad@unizar.es

**Table 5:**  Table tennis articles selected.

|  | Table tennis | | | | | | | | |
| --- | --- | --- | --- | --- | --- | --- | --- | --- | --- |
| Author | **Year** | **N** | **Sex** | **Age (±SD)** | **I** | **LA (±SD) mmol/L** | **VO_2max_ (±SD) ml/kg/min** | **VO_2_ (±SD) ml/kg/min** | **HR (±SD)**  **bpm** |
| Martin et al | 2015 | 8 | M | 23.3(±4.8) | SM | 4.7(±2.2) (O/D)  4.0(±2.2) (O/O) | NRI | NRI | 146.0(±5.9) (O/D)  139.9(±9.0) (O/O) |
| Milioni et al | 2018 | 15 | M | 21(±4) | SM | 1.4(±0.4) (O)  1.6(±0.4) (OF)  1.2(±0.4) (AR) | 45.5(±5.3) (O)  42.9(±4.2) (OF)  48.1(±5.8) (AR) | 29.5(±3.8) (O)  30.7(±4.7) (OF) 28.5(±3.0) (AR) | 142(±11) (O)  145(±11) (OF)  138(±12) (AR) |
| Picabea et al | 2021 | 21 | M | 21.86(±8.34) | SM | NRI | NRI | NRI | 103.99(±15.09) |
| Pradas de la Fuente et al | 2015b | 18 | M | 25.3(±4.3) | SM | 1.27(±0.19) | 52.2(±6.84) | NRI | 135(±7.9) |
| Pradas et al | 2021b | 24  24 | M  W | 25.3(±4.0) (M)  22.3(±3.8) (W) | SM | 1.8(±0.3) (M)  1.5(±0.2) (W) | 53(±6.03) (M)  44.2(±5.6) (W) | NRI | 138.7(±12.08) (M)  137.2(±6.03) (W) |
| Shieh et al | 2010 | 60 | M | 22.6(±2.58) | SM | NRI | 42.1(±6.4) | 36.8(±13.2) (A)  35.6(±18.4) (B) | NRI |
| Torre et al | 2022 | 24  24 | M  W | 25.3(±4.0) (M)  22.3(±3.8) (W) | SM | 1.82(±0.3) (M)  1.5(±0.2) (W) | 53(±6.03) (M)  44.2(±5.6) (W) | NRI | 138.7(±3.6) (M)  137.2(±4.7) (W) |
| Zagatto et al | 2016 | 11 | M | 22(±3) | SM | 1.89(±0.73) | 45.2* | 29.4(±4.8) | 138.1(±13.6) |

N=number of subjects; I=intervention; SM= simulated match; OM=official match; RI=does not record information OD=offensive vs defensive matches; OO= offensive vs offensive matches; A=elite players; B=amateur players; O=overall; OF=offensive; AR=all-round; M=men; W=women; *=absolute values, no standard deviation; NRI=does not record information.
