# Supplementary material for: Physiological demands of racket sports: a systematic review
Source: Front Psychol. 2023 Mar 30;14:1149295. doi: 10.3389/fpsyg.2023.1149295 (PMC10101231; doi:10.3389/fpsyg.2023.1149295)
Supplement: Supplementary file 6 [file Table_6.docx]

Supplementary Material

***PHYSIOLOGICAL DEMANDS OF RACKET SPORTS***

***A SYSTEMATIC REVIEW***

María Pía Cádiz Gallardo, Francisco Pradas de la Fuente*, Alejandro Moreno-Azze, Luis Carrasco Páez.

*** Correspondence:** franprad@unizar.es

**Table 6.** Tennis articles selected.

|  | Tennis | | | | | | | | | |  |
| --- | --- | --- | --- | --- | --- | --- | --- | --- | --- | --- | --- |
| Author | | **Year** | **N** | **Sex** | **Age (±SD)** | **I** | **LA (±SD)**  **mmol/L** | **VO_2max_ (±SD) ml/kg/min** | **VO_2_ (±SD) ml/kg/min** | **HR (±SD)**  **bpm** | |
| Baiget et al | | 2015 | 20 | M | 18.0(±1.2) | SM | NRI | 58.0(±4.6) | 29.9(±3.7) | 138(±15) | |
| Gomez et al | | 2011 | 2 | M | 20(P1) *  26(P2) * | SM | (P1) * (P2) *  1°=2.8 1°=3.2  2°=3.2 2°=3.7  3°=4.1 3°=5.0  4º=2.6 4°=2.2 | 57(P1)  53(P2) | NRI | (P1) * (P2) *  1°=137 1°=128  2°=144 2°=138  3°=152 3°=143  4º=154 4°=146 | |
| Hoope et al | | 2020 | 12 | W | 25(±5) | SM | 1.5 -2.3 * | 40.9(±4.3) | NRI | 162 * | |
| Kilit et al | | 2016 | 10 | M | 22.2(±2.8) | SM | NRI | 49.8(±1.2) | 26.6(±2.7) | 142.7(±9.5) | |
| Martin et al | | 2015 | 6 | 4 M  2 W | 22(± 2.9) | SM | 5.7(± 1.8)  3.6(±1.2) | NRI | NRI | 144(±12)  141(± 9) | |
| Mendez-Villanueva et al | | 2010 | 8 | M | 27.0(± 4.4) | SM | 4.46(±2.4)  3.0(±1.3) | NRI | NRI | NRI | |

N=number of subjects; I=intervention; SM= simulated match; OM=official match; P1=player one; P2=player two; *=absolute values, no standard deviation; M= men W= women; NRI=does not record information.
