# Supplementary material for: Physiological demands of racket sports: a systematic review
Source: Front Psychol. 2023 Mar 30;14:1149295. doi: 10.3389/fpsyg.2023.1149295 (PMC10101231; doi:10.3389/fpsyg.2023.1149295)
Supplement: Supplementary file 7 [file Table_7.docx]

Supplementary Material

***PHYSIOLOGICAL DEMANDS OF RACKET SPORTS***

***A SYSTEMATIC REVIEW***

María Pía Cádiz Gallardo, Francisco Pradas de la Fuente*, Alejandro Moreno-Azze, Luis Carrasco Páez.

*** Correspondence:** franprad@unizar.es

**Table 7.** Badminton articles selected.

|  | Badminton | | | | | | | | | |  |
| --- | --- | --- | --- | --- | --- | --- | --- | --- | --- | --- | --- |
| Author | | **Year** | **N** | **Sex** | **Age (±SD)** | **I** | **LA (±SD) mmol/L** | **VO_2max_ (±SD) ml/kg/min** | **VO_2_ (±SD) ml/kg/min** | **HR (±SD)**  **bpm** | |
| Abudalli et al | | 2019 | 21 | M | 23.2(±3.6) | OM | NRI | NRI | NRI | 157.1(±13.9) | |
| Bisschoff et al | | 2016a | 22 | M | 23.3(±3.9) | OM | NRI | NRI | NRI | 167(±17) | |
| Bisschoff et al | | 2016b | 22 | M | 23.3(±3.9) | OM | NRI | NRI | NRI | 166.76(±13.84) | |
| Bisschoff et al | | 2018 | 22 | M | 23.3(±3.9) | OM | NRI | NRI | NRI | 166.76(±13.84) | |
| Chen et al | | 2011 | 10 | M | 20.6(±1.4) | OM | 4.6(±0.4) (N) 5.3(±0.7) (O) | NRI | NRI | 178.9(±1.8) (N)  182.6(±2.7) (O) | |
| Deka et al | | 2017 | 14 | M | 35.9(±6.6) | SM | 10.11(±4.9) | 45.2(±8.7) | 34.4(± 5.8) | 167(±9.4) | |
| Savarirajan et al | | 2016 | 11 | 10 M  1 W | 21.8(±3.26) | SM | NRI | NRI | NRI | 162-187* | |

N=number of subjects; I=intervention; SM= simulated match; OM= official match; N=values at stake according to game rules 3x15; O= values according to game rules 3x21; M= men; W= women; *=absolute values, no standard deviation; NRI=does not record information.

**Additional references:**

Abdullahi, Y., Coetzee, B., and van den Berg, L. (2019) Relationships between results of an internal and external match load determining method in male, singles badminton players. *Journal of strength and conditioning research*, 33(4), 1111–1118. <https://doi.org/10.1519/JSC.0000000000002115>

Bisschoff, C., Coetzee, B., Esco, M. (2016a). Relationship between autonomic markers of Heart Rate and Subjective Indicators of Recovery Status in Male, Elite Badminton Players. *Journal of sports science & medicine*, 15(4), 658–669.

Bisschoff, C., Coetzee, B., Esco, M. (2016b) Relationship between heart rate, heart rate variability, heart rate recovery and global positioning system determined match characteristics of male, elite, African badminton player. *International Journal of Performance Analysis in Sport,* 16, 881-897. https://doi.org/ [10.1080/24748668.2016.11868936](http://dx.doi.org/10.1080/24748668.2016.11868936)

Bisschoff, C., Coetzee, B., Esco, M. (2018) Heart rate variability and recovery as predictors of elite, African, male badminton players performance levels. *International Journal of Performance Analysis in Sport*. [https://doi.org/18. 1-16. 10.1080/24748668.2018.1437868](https://doi.org/18.%201-16.%2010.1080/24748668.2018.1437868)

Savarirajan, R. (2016) Result of heart rate, playing time and performance of tamilnadu badminton senior ranking players. *International Journal of Sports Sciences and Fitness*, 6(1), pp. 43–57.
