# Supplementary material for: Physiological demands of racket sports: a systematic review
Source: Front Psychol. 2023 Mar 30;14:1149295. doi: 10.3389/fpsyg.2023.1149295 (PMC10101231; doi:10.3389/fpsyg.2023.1149295)
Supplement: Supplementary file 8 [file Table_8.docx]

Supplementary Material

***PHYSIOLOGICAL DEMANDS OF RACKET SPORTS***

***A SYSTEMATIC REVIEW***

María Pía Cádiz Gallardo, Francisco Pradas de la Fuente*, Alejandro Moreno-Azze, Luis Carrasco Páez.

*** Correspondence:** franprad@unizar.es

**Table 8.**  Padel articles selected.

|  | Padel | | | | | | | | | |  |
| --- | --- | --- | --- | --- | --- | --- | --- | --- | --- | --- | --- |
| Author | | **Year** | **N** | **Sex** | **Age (±SD)** | **I** | **LA (±SD)**  **mmol/L** | **VO_2max_ (±SD)**  **ml/kg/min** | **VO_2_ (±SD)**  **ml/kg/min** | **HR (±SD)**  **bpm** | |
| Carbonell Martínez et al | | 2017 | 9 | W | 32.8(±12.3) | OM | NRI | NRI | NRI | 150(±8.6) | |
| Castillo-Rodriguez et al | | 2014 | 24 | M | 28.70(±6.76) | OM | 2.87(±1.48) | NRI | NRI | 149.1(±18.27) | |
| García et al | | 2017 | 8 | M | 22.48(±1.12) | SM | NRI | 51.15(±5.73) | NRI | 126.78(±10.4) | |
| Pradas de la Fuente et al | | 2015a | 6 | W | 28.2(±0.6) | SM | 1.83 | 47.33(±4.57) | NRI | 151(±8.1) | |
| Ramón-Llin et al | | 2018 | 14 | M | 25.4(±3.8) (SV)  31.14(±5.9) (PPT) | OM | NRI | NRI | NRI | 159.1(SV)*  153.7(PPT)* | |
| Roldán-Márquez et al | | 2022 | 24 | M | 28.708(±6.76) | SM | 2.72(±1.38) (W)  2.98(±1.50) (L) | NRI | NRI | 144.2(±19.1) (W)  154.1(±15.6) (L) | |

N=number of subjects; I=intervention; SM= simulated match; OM=official match; SV=Valencian National Team; PPT=Padel Pro Tour; W=match winner; L=match loser; M= men; F= women; *=absolute values, no standard deviation; NRI=does not record information.

Carbonell Martínez, J. A., Ferrándiz Moreno, J. y Pascual Verdú, N. (2017) Análisis de la frecuencia cardíaca en el pádel femenino amateur (Analysis of heart rate in amateur female padel), Retos. 204-207. 10.47197/retos.v0i32.56040
